# Health literacy and refugees’ experiences of the health examination for asylum seekers – a Swedish cross-sectional study

**DOI:** 10.1186/s12889-015-2513-8

**Published:** 2015-11-23

**Authors:** Josefin Wångdahl, Per Lytsy, Lena Mårtensson, Ragnar Westerling

**Affiliations:** Social Medicine, Department of Public Health and Caring Science, Uppsala University, Uppsala Science Park, Box 564, 751 22 Uppsala, Sweden; Institution of Department of Neuroscience and Physiology/Occupational Therapy, University of Gothenburg, Box 455, SE 405 30 Göteborg, Sweden

**Keywords:** Health literacy, Refugees, Health examination, Health promotion, Sweden, S-FHL, HLS-EU-Q16

## Abstract

**Background:**

The purpose of the health examination for asylum seekers in most countries is to identify poor health in order to secure the well-being of seekers of asylum and to guarantee the safety of the population in the host country. Functional health literacy is an individual’s ability to read information and instructions about health and to function effectively as a patient in the health system, and comprehensive health literacy is an individual’s competence in accessing, understanding, appraising and applying health information. Little is known about refugees’ health literacy and their experiences of the health examination for asylum seekers. The purposes of the study were to investigate refugees’ experiences of communication during their health examination for asylum seekers and the usefulness of that examination, and whether health literacy is associated with those experiences.

**Methods:**

A cross-sectional study was made among 360 adult refugees speaking Arabic, Dari, Somali or English. Health literacy was measured using the Swedish Functional Health Literacy Scale and the short European Health Literacy Questionnaire. Experiences of communication and the usefulness of the health examination were measured in several questions. Associations were sought using univariate and multivariate statistical models.

**Results:**

In the health examination for asylum seekers, a poor quality of communication was experienced by 36 %, receiving little information about health care by 55 %, and receiving little new knowledge by 41 % and/or help by 26 %. Having inadequate as compared to sufficient comprehensive health literacy was associated with the experience of a poorer quality of communication (OR: 9.64, CI 95 %: 3.25–28.58) and the experience of receiving little valuable health care information (OR: 6.54, CI 95 %: 2.45–17.47). Furthermore, having inadequate as compared to sufficient comprehensive health literacy was associated with the experience of not receiving new knowledge (OR: 7.94, CI 95 %: 3.00–21.06) or receiving help with health problems (OR: 8.07, 95 % CI: 2.50–26.07. Functional healthy literacy was not associated with experiences of HEA.

**Conclusion:**

Refugees’ experiences indicate that a low level of comprehensive health literacy can act as a barrier to fulfilling the purposes of the health examination for asylum seekers. Comprehensive health literacy seems to be of greater importance in that context than functional health literacy.

**Electronic supplementary material:**

The online version of this article (doi:10.1186/s12889-015-2513-8) contains supplementary material, which is available to authorized users.

## Background

The overall purpose of health examinations for asylum seekers (HEA) in most countries is to identify poor health in order to secure the well-being of seekers of asylum and to guarantee the safety of the population in the host country [1]. Another purpose of the HEA in many countries is to provide information about the health system in the new country in order to increase refugees’ access to health care [2–4].

Little is known about refugees’ health literacy (HL) and their experiences of the health examination for asylum seekers (HEA). Health literacy is a key determinant for health and empowerment [5]. Roughly, two forms of health literacy dominate in the scientific literature: functional and comprehensive health literacy [6, 7]. Functional health literacy (FHL) comprises individuals’ ability to read information and instructions about health that are needed to function effectively as a patient in the health system [6, 7]. Comprehensive health literacy (CHL), has been defined by Sorensen et al. [6] as:

“…links to literacy and entails people’s knowledge, motivation and competences to access, understand, appraise, and apply health information in order to make judgments and decisions in everyday life concerning healthcare, disease prevention and health promotion to maintain or improve quality of life during the life course” (page 3).

Functional HL is a more fundamental phenomenon as compared with comprehensive HL, which includes a variety of skills, not only related to people’s physical functions but also to communicative and interaction skills.

One group with a high proportion of people with limited overall HL is migrants. In studies performed in Canada [8] about 80 % of migrants had limited FHL, which is in line with our previous findings in Sweden that focused on refugees [9]. The only known study having assessed CHL among migrants shows that about 60 % of those had limited CHL. Communication problems and difficulties with HL are common in clinical care targeted to migrants [10–13]. Furthermore, migrants often receive less health information than others [14]. Limited language skills, different cultural views of health, and health care knowledge about health [11–13, 15, 16] and HL [17–20] may explain some of the communication problems. From a public health perspective, communication problems are serious as they limit access to health care and information. This is seen as an explanatory factor of many migrants’ poor health [13, 21].

One vulnerable group of migrants in terms of health is refugees [16, 21, 22]. In 2011, 10.4 million migrants were classified as refugees [23], i.e. as persons who have fled from and/or cannot return to their country for a well-founded fear of persecution, including war or civil conflict [24]. The most common countries of origin were Afghanistan (2.7 million), Iraq (1.4 million) and Somalia (1.1 million).

Many refugees’ first contact with health care and health information in the new country is when they participate in an HEA, which is provided in most countries that accept refugees [1, 2]. However, refugees’ experiences of communication of health information during the HEA and about its usefulness are thus far not known. Important information, good communication and interpersonal relations between health care receivers and providers are viewed as important for the quality of health care [25, 26]. Examples of behavior among health care providers associated with good communication are: listening, encouraging questions, talking on an appropriate level, checking understanding, and addressing the health receiver’s problems [27, 28]. Health care receivers’ experiences of health care indicate the effectiveness of the health care—the extent to which a treatment or service is consistent with the health care receiver’s expectations in the delivery and outcome of the health care visit [25]. Health care receivers’ perspectives of the quality of care are infrequently addressed but are important complementary indicators to the more common indicators, such as the medical outcome of care (ibid.).

From an ethical perspective, it is important to understand refugees’ perspectives of whether HEA meets individuals’ needs or if it mainly addresses a societal need. Limited HL can threaten an individual’s autonomy and thus limit chances of getting appropriate health care [29]. In the context of HEA, limited HL may result in failure to identify health problems and in participants not getting treatments and information their medical situation calls for. Knowledge about associations between HL and refugees’ experiences of HEA is lacking. This knowledge could indicate whether HL is important in the context of the HEA.

### Purpose

The purposes of the study were to investigate refugees’ experiences of communication during their HEA and the usefulness of that examination, and whether HL is associated with those experiences.

We hypothesized that:

- Refugees with inadequate and problematic HL experience more communication problems during HEA as compared to those with sufficient HL.
- Refugees with inadequate and problematic HL experience HEA as less useful as compared to those with sufficient HL.

## Method

### Study design and setting

The study had a cross-sectional design and was carried out in 2013 in four counties, in different demographic and geographic areas of Sweden. All asylum seekers in Sweden must by law be offered a health examination, unless it is clearly unnecessary, free of charge [30]. The term asylum seeker in this article is used for someone whose refugee claim has yet to be definitely evaluated. The HEA can be carried out either before or after having received a permanent resident permit as a seeker of asylum. The HEA must include a dialogue about the participant’s earlier physical and psychological health and vaccinations and questions of importance from the point of view of infection control [4]. Physical examinations and clinical tests are based on what emerges in the dialogue. The participants should further be given information about their right to health care and how to access health care in Sweden.

Ethics approval was sought at the regional Ethical Committee of Clinical Investigation in Uppsala, Sweden, registration number 2012:506, but a committee judgment was not deemed necessary or applicable according to Swedish law, since data collection was performed anonymously, leaving no possibility of individual identification.

### Study population

The target group was adult refugees in schools offering Swedish for immigrants (SFI). Inclusion criteria were: respondents eligible on the day of the data collection; speakers of Arabic, Somali, Dari or English; born outside a Nordic country or the EU; and having received a permanent resident permit as a seeker of asylum. This means that refugees were asked about their participation in a HEA that was done either before or after having received a permanent resident permit as a seeker of asylum. Of 455 eligible SFI students, 360 fulfilled the inclusion criteria as well as participated in HEA and, thus formed the study population in the present study.

### Data collection

A strategic selection of SFI schools was based on the number of people who had received resident permits as asylum seekers in each municipality in 2012, in each county [9]. Eligible SFI schools with more than 30 students were selected from each municipality in the four counties. If more than one school in a municipality fulfilled the criterion, a school was randomly drawn. Nineteen schools agreed to participate and were visited to collect data.

A team consisting of a researcher (first author) and a number of language supporters visited each school to collect data. The SFI students were verbally informed about the project and voluntary participation. Eligible participants were grouped on the basis of their native language. They were then informed about the project, again in their native language, and asked for verbal consent. Those who consented to participate were given the questionnaire, which was completed on site. Twenty one percent of the participants (70 out of 360) who had difficulty reading or writing were assisted by language supporters that read and supported them in filling in the questionnaire.

#### Material and analysis

Data were collected using a questionnaire with 60 questions that focused on health, HL and experiences of HEA. The questions that concerned HEA were based on the results of an earlier qualitative explorative pilot study (Åkerman E and Wångdahl J, 2014, “Unpublished observation”). The questionnaire was translated by language supporters into Arabic, Somali, Dari and English following guidelines for the translation of instruments [31].

### Characteristics

The socio-demographic and health-related characteristics under examination were sex, age, country of birth, education level, self-assessed general state of health and years of having had a resident permit. Background questions related to the HEA were whether the individual had participated in an HEA in Sweden, when he or she had participated and whether support by an interpreter had been given. FHL was measured by the Swedish FHL scale (S-FHL scale) which consists of five items assessing different aspects of FHL [32] (Additional file 1).

When analysing the data, an overall level for FHL was calculated for each respondent [9]. Persons responding “never” or “seldom” to all items were categorized as having sufficient HL. Persons scoring “often” or “always” to one or more of the five items were categorised as having inadequate HL. The rest, those who responded “sometimes” to at least one item and not “often” or “always” to any items, were categorised as having problematic HL. The cut-offs used when dividing the respondents into the three groups were based on definitions describing the abilities needed for sufficient FHL, i.e. basic skills in reading information and instructions about health [7]. Those lacking any basic skills were classified as having inadequate FHL, those having all the basic skills were classified as having sufficient FHL and those in between were classified as having problematic FHL.

CHL was measured by a slightly modified Swedish version of the short European HL questionnaire (HLS-EU-Q16) [9, 33] (Additional file 2). This focused on four HL dimensions: ability to access, understand, appraise and apply health information. An overall HLS-EU-Q16 index (CHL) was calculated in three steps when the data were analysed, following the manual for the instrument [33]. The response categories were first dichotomized [9]. The responses “fairly easy” and “very easy” were put together into one category, which was given the value of 1, the responses “fairly difficult” and “very difficult” were put together into one category which was given the value of 0, and the response “don’t know” was treated as missing. Second, a sum score of the response values was calculated and divided into three categories: sufficient CHL if there were 13–16 score points, problematic CHL if there were 9–12 score points or inadequate CHL if there were 0–8 score points.

In the case of missing values in two or more items of the HLS-EU-Q16 scale*,* any missing value on an item was substituted with “difficult” and a new CHL value was calculated, i.e. all participants thereafter had a CHL value (CHL^m^). CHL^m^ was used in analyzing associations between experiences of HEA and HL, and what could predict poor experiences of communication and the usefulness of HEA. The treatment of missing values was chosen in a dialogue with Florian Röthlin, statistician at the Ludwig Boltzman Institute for Health Promotion research (“personal communication”, October 28, 2014), who worked with the development of the HLS-EU-Q16. The multiple imputations method was considered, but this would have changed the scoring algorithm (ibid). Thirty six percent (123 out of 360) of the participants had an incomplete CHL value; thus the CHL^m^ was calculated for those participants.

The S-FHL scale and HLS-EU-Q16 measure quite different aspects of HL. The S-FHL scale is focusing on the individual’s abilities to read and understand written health information. The HLS-EU-Q16 focuses on cognitive abilities in order to access, understand, appraise and apply oral health information as well. In dichotomizing FHL/CHL, inadequate and problematic FHL/CHL were merged into limited FHL/CHL. The scales measuring health literacy have been validated by carrying out cognitive interviews concerning the content with refugees comparable with the study group.

### Experiences of communication and the usefulness of HEA

Experiences of communication and the usefulness of HEA were measured in four dependent variables (Additional file 3). *Quality of communication,* measured the experience of communication, used in this study as an umbrella term for different aspects of communication. *Quality of communication* was measured in four questions in an attempt to omit different aspects of communication. The response alternatives “no”, “partly” and “yes” were assigned values from 1 to 3, respectively, yielding an index with a maximum total score of 12. The usefulness of HEA was measured by three dependent variables: *receiving health care information*, *receiving new knowledge* and *receiving help. Receiving health care information* was measured in three questions. The response alternatives “no” and “yes” there were values from 1 to 2, yielding an index with a maximum total score of 6. *Receiving new knowledge* and *receiving help* were measured using one question each. The response alternatives “no”, “partly” and “yes” for those dependent variables were assigned values from 1 to 3, respectively, yielding indexes with a maximum total score of 3. The responses of participants who answered “no” or “don’t know” to the questions on participation in HEA were not included in the analysis. Values in the response categories “don’t remember” in the questions on the quality of communication and usefulness of HEA were treated as missing.

### Statistical analysis

The statistical analyses used SPSS version 21.0 (Chicago, IL, USA). Chi square tests or Fisher’s exact tests when the expected frequency in each cell was lower than 5, were used to compare proportions of experiences of various aspects of HEA in groups with different FHL and CHL levels. Binary logistic regressions were done to calculate the crude odds ratios for the effect of socio-demographic factors, support of an interpreter and HL levels in the four dependent variables. Cronbach’s alphas were calculated to explore the internal consistency of the questions used for *quality of communication* and *receiving health care information*. The internal consistency was satisfactory (Cronbach’s alpha = 0.79 and 0.71, respectively).

Multiple binary logistic regression was used to investigate the association between FHL, CHL^m^ and the four dependent variables focusing on different experiences of HEA, adjusting for sex, age, education, country of origin and support of an interpreter. The multivariate model (Tables 3 and 4) included sex, age, education, country of birth, interpreter support, FHL and CHL^m^. Results are presented as crude odds ratios and adjusted odds ratios (OR) with 95 % confidence intervals (CI) and *p*-values. A *p*-value of < 0.05 was considered statistically significant, and all analyses were two-sided. The dichotomized cut-offs for the dependent variables in the logistic regression analyses were suggested by the data. Each dependent variable was dichotomized into a higher (median and above) and a lower (below median) group based on the median.

In addition to the binary logistic regression analysis including CHL^m^ (all participants), logistic regressions were performed with CHL, only including participants having a valid CHL from the beginning. The results for HL were not changed in any major sense.

### Sensitivity analyses

Alternative cut-offs for the dichotomous dependent variables were tested to identify the robustness of the results of the multivariate analysis. The cut-off levels for *quality of communication* and *receiving health care information* were moved downwards one step, and the cut-off levels for *receiving new knowledge* and *receiving help* were moved one step upwards. Thus, the results for HL were not changed in any major sense.

## Results

### Characteristics

The study population was heterogeneous (Table 1). There were slightly more men than women, and the average age was 35.4 years (S.D. 10.5). Most participants were born in Somalia, Iraq, Syria or Afghanistan, and the rest of the participants came from a variation of countries in Africa and Asia. Most had studied 7 years or more, almost half had very poor to fair self-reported health and the majority had received a resident permit in Sweden 1 to 2 years previously. Reporting limited FHL was more common than reporting limited CHL. Most participants had received an HEA less than a year before the study, and most were supported by an interpreter at the visit.

**Table 1:** Distribution of characteristics of the study population^a^

Variables (*n* = 360)	Number	Total distribution (%)
Gender (number = 354)		
Men	184	52.0
Women	170	48.0
Age (*n* = 325)		
18–24	61	18.8
25–44	201	61.8
45 years or older	63	19.4
Country (*n* = 310)		
Afghanistan	30	9.7
Iraq	63	20.3
Somalia	94	30.3
Syria	66	21.3
Other country	57	18.4
Education (*n* = 355)		
None	47	13.2
1–6 years	84	23.7
7–12 years	122	34.4
More than 12 years	102	28.7
Functional health literacy (*n* = 315)		
Inadequate	190	60.3
Problematic	59	18.7
Sufficient	66	21.0
Comprehensive health literacy (*n* = 237)		
Inadequate	66	27.8
Problematic	77	32.5
Sufficient	94	39.7
Comprehensive health literacy^m^ (*n* = 360)		
Inadequate	151	41.9
Problematic	110	30.6
Sufficient	99	27.5
Self-assessed health (*n* = 351)		
Very poor	12	3.4
Poor	36	10.3
Fair	107	30.5
Good	94	26.8
Very good	102	29.1
Years of residential permit (*n* = 321)		
Less than 1 year	21	6.5
1–2 years	209	65.1
More than 2 years	91	28.3
Time since participating in a health examination for asylum seekers (*n* =354)		
Less than a year ago	178	50.3
1–2 years ago	87	24.6
More than 2 years ago	89	25.1
Support of interpreter (*n* = 318)		
Yes	241	75.8
No	77	24.2

^a^Missing data not included

### Experiences of HEA

A considerable proportion of the participants experienced that they received little health care information during HEA and that the quality of the communication was low (Table 2). An even higher proportion experienced that they were not informed about their right to health care or where to go if they were mentally ill. Furthermore, many of the participants experienced that they did not receive any new knowledge or help during the HEA.

**Table 2:** Proportion of respondents with non-good experiences of the communication quality and the usefulness of the health examination^a^

	**Total study population**		**Comprehensive health literacy** ^**ab**^		
	**Respondents/ response category**	**Inadequate**	**Problematic**	**Sufficient**	***p*** **-value**
**Response categories**	**Number (%)**	199 **Number (%)**	141 **Number (%)**	115 **Number (%)**	
**Understood what was being told (** ***n*** **= 329)**					**<0.001** ^**c**^
No	20 (6.1)	**16 (11.9)**	**3 (2.9)**	**1 (1.1)**	
Partly	69 (21.0)	**34 (25.2)**	**18 (17.5)**	**17 (18.2)**	
Yes	240 (72.9)	**85 (63.0)**	**82 (79.5)**	**73(80.2)**	
**Could talk about health problems (** ***n*** **= 321)**					**<0.001** ^**c**^
No	49 (15.3)	**32 (24.1)**	**12 (12.2)**	**5 (5.6)**	
Partly	22 (6.9)	**16 (12.0)**	**4 (4.1)**	**2 (2.2)**	
Yes	250 (77.9)	**85 (63.9)**	**82 (83.7)**	**83 (92.2)**	
**Could ask questions (** ***n*** **= 318)**					**<0.001** ^**c**^
No	55 (17.3)	**33 (26.2)**	**12 (12.0)**	**10 (10.9)**	
Partly	53 (16.7)	**28 (22.2)**	**16 (16.0)**	**9 (9.8)**	
Yes	210 (66.0)	**65 (51.6)**	**72 (72.0)**	**73 (79.3)**	
**Received answers to questions asked (** ***n*** **= 299)**					**<0.001** ^**c**^
No	52 (17.4)	**31 (26.3)**	**10 (11.0)**	**11 (12.2)**	
Partly	54 (18.1)	**30 (25.4)**	**19 (20.9)**	**5 (5.6)**	
Yes	193 (64.5)	**57 (48.3)**	**62 (68.1)**	**74 (82.2)**	
**Quality of communication (** ***n*** **= 275)**					
Low quality	100 (27.8)	**60 (56.6)**	**25 (28.7)**	**15 (18.3)**	**<0.001** ^**c**^
High quality	175 (63.6)	**46 (43.3)**	**62 (71.3)**	**67 (8.7)**	
**Received information about**					
**… asylum seekers’ rights to health (** ***n*** **= 306)**					
No	137 (44.8)	**78 (61.4)**	**40 (44.0)**	**19 (21.6)**	**<0.001** ^**c**^
Yes	169 (55.2)	**49 (38.6)**	**51 (56.0)**	**69 (78.4)**	
**…where to go if one becomes sick in Sweden (** ***n*** **= 324)**					
No	76 (23.5)	**49 (36.6)**	**14 (14.3)**	**13 (14.1)**	**<0.001** ^**c**^
Yes	248 (76.5)	**85 (63.4)**	**84 (85.7)**	**79 (85.9)**	
**…where to go in Sweden if one feels mentally unwell (** ***n*** **= 306)**					
No	169 (55.2)	**85 (70.2)**	**52 (53.1)**	**32 (36.8)**	**<0.001** ^**c**^
Yes	137 (44.8)	**36 (29.8)**	**46 (46.9)**	**55 (63.2)**	
**Received little health care information (** ***n*** **= 206)**					
Little	113 (31.4)	**67 (63.2)**	**30 (37.0)**	**16 (20.3)**	**<0.001** ^**c**^
Much	153 (57.5)	**39 (36.8)**	**51 (63.0)**	**63 (79.7)**	
**Received new knowledge (** ***n*** **= 301)**					**<0.001** ^**c**^
No	124 (34.4)	**73 (60.8)**	**35 (36.5)**	**16 (18.8)**	
Partly	63 (20.9)	**21 (17.5)**	**24 (25.0)**	**18 (21.2)**	
Yes	114 (37.9)	26 (21.7)	37 (38.5)	51 (60.0)	
**Received help (** ***n*** **= 316)**					**<0.001** ^**c**^
No	83 (23.1)	**52(41.3)**	**22 (21.8)**	**9 (10.1)**	
Partly	99 (31.3)	**44 (34.9)**	**36 (35.6)**	**19 (21.2)**	
Yes	134 (42.4)	**30 (23.8)**	**43 (42.6)**	**61(68.5)**	

^a^Missing data not included

^b^Modified CHL value

^c^Chi square test; *n* (%) = number and proportions of participants; significant differences *p* < 0. 05 are printed in bold

### Associations between HL and experiences of HEA

No associations were found between FHL levels and any variable dealing with experiences of communication during HEA and the usefulness of HEA (Additional file 4). However, associations were found between CHL levels and all variables regarding experiences of the quality of communication and usefulness of HEA (Table 2). In subgroups of participants with limited CHL, higher proportions of participants experienced that they could not ask questions and/or had received answers to questions they had asked than in the subgroup with participants that had sufficient CHL. A similar pattern was found in the associations between CHL and experiences of the usefulness of the HEA.

### Factors associated with the experiences of HEA

In the fully adjusted model, inadequate and/or problematic FHL were not significant predictors of low quality of communication during HEA or of having experienced HEA as less useful Tables 3 and 4). However, in the fully adjusted model, participants with inadequate and/or problematic CHL had increased odds of having experienced a poor quality of communication during HEA or having experienced HEA as less useful (Tables 3 and 4). It was more likely that those with inadequate CHL experienced poor quality of communication during HEA, received little health care information, experienced receiving little new health knowledge and/or experienced that they received no help with health problems during HEA, compared with those with sufficient CHL.

**Table 3:** Odds ratios of having experienced poor quality of communication and having received little health information during the health examination

	ORs of having experienced poor quality of communication					ORs of having received little health care information				
Variables	Crude^a^ OR (95 % CI)			OR^b^ (95 % CI)		Crude^a^ OR (95 % CI)			OR^b^ (95 % CI)	
	Number of respondents/ analysis			Number of respondents/ analysis:168		Number of respondents/ analysis			Number of respondents/ analysis:164	
Sex	270					262				
Men		1		1			1		1	
Women		1.06	(0.65–1.74)	1.34	(0.61–2.92)		0.87	(0.53–1.42)	0.57	(0.26–1.25)
Age	247					240				
18–24		1		1			1		1	
25–44		1.25	(0.62–2.51)	1.08	(0.43–2.74)		1.02	(0.51–2.01)	0.81	(0.33–2.00)
44-		0.98	(0.42–2.29)	1.14	(0.35–3.70)		0.61	(0.27–1.39)	0.74	(0.24–2.23)
Education	271					263				
0–6 years		0.71	(0.42–1.21)	1.11	(0.47–2.63)		0.78	(0.47–1.30)	1.14	(0.51–2.58)
≤ 7 years		1		1			1		1	
Country	235					222				
Iraq		1		1			1		1	
Other country		1.27	(0.53–3.02)	1.40	(0.40–4.95)		2.03	(0.85–4.83)	3.03	(0.85–10.71)
Syria		0.72	(0.30–1.73)	0.60	(0.19–1.91)		1.03	(0.44–2.42)	0.72	(0.24–2.15)
Afghanistan		1.33	(0.47–3.78)	0.66	(0.15–2.88)		2.10	(0.74–5.94)	0.90	(0.22–3.69)
Somalia		0.92	(0.42–2.03)	0.61	(0.19–1.98)		1.27	(0.58–2.76)	0.99	(0.34–2.91)
Support by interpreter	250					243				
Yes		1		1			1		1	
No		0.36	(0.20–0.66)**	2.35	(1.00–5.48)*		3.16	(1.71–5.83)***	2.49	(1.08–5.75)*
FHL	250					244				
Inadeqate		1.50	(0.78–2.89)	0.81	0.25–2.67)		1.36	(0.71–2.61)	0.74	(0.25–2.22)
Problematic		1.14	(0.50–2.50	0.86	(0.22–3.29)		1.20	(0.53–2.74)	0.60	(0.17–2.17)
Sufficient		1		1			1		1	
CHL^m^	275					266				
Inadequate		5.83	(2.96–11.49)***	9.64	(3.25–28.58)***		6.76	(3.44–13.30)***	6.54	(2.45–17.47)***
Problematic		1.80	(0.87–3.73)	1.84	(0.63–5.37)		2.32	(1.14–4.72)*	3.39	(1.27–9.05)*
Sufficient		1		1			1		1	

*Significant at *p* < 0.05; **Significant at *p* < 0.01; ***Significant at *p* < 0.001

^a^Crude OR for explanatory factors considered

^b^OR for included explanatory factors: sex, age, education, country, support of interpreter, FHL and CHL

**Table 4:** Odds ratios of not having received any new knowledge or help during the health examination

	ORs of not having received any new knowledge					ORs of not having received any help				
Variables	Crude^a^ OR (95 % CI)			OR^b^ (95 % CI)		Crude^a^ OR (95 % CI)			OR^b^ (95 % CI)	
	Number of respondents/analysis			Number of respondents/analysis: 190		Number of respondents/analysis			Number of respondents/analysis: 196	
Sex	295					310				
Men		1		1			1		1	
Women		0.66	(0.41–1.05)	0.33	(0.16–0.68)**		0.67	(0.40–1.12)	0.58	(0.27–1.25)
Age	270					284				
18–24		1		1			1		1	
25–44		1.40	(0.73–2.68)	1.30	(0.55–3.06)		1.33	(0.65–2.75)	1.39	(0.55–3.52)
44-		0.89	(0.40–2.00)	0.82	(0.28–2.40)		0.96	(0.39–2.36)	0.91	(0.26–3.16)
Education	296					311				
0–6 years		1.45	(0.83–2.19)	1.42	(0.66–3.06)		1.54	(0.91–2.62)	2.28	(0.98–5.33)
≤ 7 years		1		1			1		1	
Country	256					270				
Iraq		1		1			1		1	
Other country		1.46	(0.65–3.31)	1.28	(0.41–3.95)		1.80	(0.71–4 0.58)	1.55	(0.45–5.34)
Syria		1.14	(0.52–2.47)	0.64	(0.23–1.76)		1.31	(0.53–3.25)	1.37	(0.43–4.33)
Afghanistan		1.73	(0.68–4.41)	0.82	(0.21–3.10)		5.06	(1.83–14.01)**	2.37	(0.57–9.92)
Somalia		1.84	(0.88–3.82)	2.41	(0.85–6.80)		1.38	(0.58–3.29)	1.30	(0.40–4.27)
Support by interpreter	272					283				
Yes		1		1			1		1	
No		1.60	(0.92–2.81)	1.73	(0.81–3.69)		1.25	(0.68–2.32)	1.55	(0.69–3.48)
FHL	277					287				
Inadeqate		1.43	(0.77–2.68)	0.55	(0.20–1.54)		2.21	(1.01–4.84)*	0.80	(0.25–2.54)
Problematic		1.24	(0.57–2.67)	0.86	(0.27–2.74)		2.27	(0.91–5.66)	0.86	(0.24–3.14)
Sufficient		1		1			1		1	
CHL^m^	301					316				
Inadequate		6.70	(3.48–12.91)***	7.94	(3.00–21.06)***		6.25	(2.88–13.56)***	8.07	(2.50–26.07)***
Problematic		2.47	(1.25–4.91)**	3.71	(1.45–9.50)**		2.48	(1.07–5.71)*	3.33	(1.02–10.86)*
Sufficient		1		1			1		1	

*Significant at *p* < 0.05; **Significant at *p* < 0.01; ***Significant at *p* < 0.001

^a^Crude OR for considered explanatory factors

^b^OR for included explanatory factors: sex, age, education, country, support of interpreter, FHL and CHL

Those with problematic CHL were more likely to have experienced that they received little health care information, experienced that they received any new health knowledge, and experienced not receiving any help with health problems during HEA, compared with those with sufficient CHL. It was furthermore more likely that the participants that did not have the support of an interpreter during HEA experienced poor quality of communication and/or experienced that they received little health information, compared with those that had the support of an interpreter.

## Discussion

The main purposes of the study were to investigate refugees’ experiences of communication during HEA and the usefulness of that examination, and to investigate whether HL is associated with those experiences. A considerable number of the participants experienced some communication problems during HEA and felt that they did not fully receive health care information, new health knowledge or help with their health problems during HEA (i.e. they experienced that the HEA was not very useful). This is new knowledge, as refugees’ own experiences of the communication in the context of HEA have not been explored before. However, communication problems between patients who are migrants and health care providers in general are well known [10, 12, 13, 15, 34].

The overall high proportions of participants who experienced some communication problems and/or not having fully received health care information, new knowledge or help with their health problems are notable. This indicates that the purpose of the HEA, to identify poor health in order to offer adequate care [1, 4], is not always fulfilled. It is also notable that almost 30 % of the participants did not fully understand what they were being told. This could affect refugees’ experiences of the accessibility of health care and thus reduce autonomy in informed decision making concerning their own health care. It may also lead to inappropriate expectations and health-seeking behaviors [11], and unnecessary health problems [12, 13, 29].

The questions about what information the participants had received during HEA do not indicate whether the information had actually been delivered. However, they offer knowledge about the participants’ own experiences of having received information. This is important, as it indicates whether the information was actually received. If refugees do not understand or have the ability to use information that they are given, it could be suggested that the aim of distributing the information is lost.

The high proportion of participants that did not receive information about where to go if they felt mentally unwell is critical, according to the high prevalence of poor mental health among refugees/migrants [16, 22, 35]. Many migrants do not know how health care systems in their new country work, which could limit their use of health care [13, 21]. Bad experience of health care in general is critical because previous health care experiences can influence an individual’s future expectations of health care [26], access to health care [21, 36] and care-seeking behavior [37]. The bad experiences of the HEA, which for many migrants is the first contact with the health care system in the new country, could therefore also have negative consequences for refugees’ future health care and health.

Having experienced a poor quality of communication and/or HEA as less useful was more common among participants with limited CHL as compared to those with sufficient CHL. However, no associations in those experiences were found between participants with different FHL. This means that the hypothesis that people with inadequate or problematic HL experience more communication problems during HEA and experience HEA as less useful, compared with those with sufficient HL, is true for CHL but not for FHL. Communication problems have previously been observed between health care receivers with limited HL and health care providers [17, 19]. Furthermore, it has been found that CHL may be of greater importance for the quality of communication during health care encounters than FHL [19, 20]. It may be regarded as logical that those with limited CHL experienced HEA as less useful, as they also had higher odds of having experienced communication problems and having received little health care information.

Communication problems can reduce the possibility to identify health problems and to meet the individual’s needs for treatment and information [38]. This may in turn lead to experiences of not having received any new knowledge or help with health problems.

The FHL skills may be understood as concrete and observable, while the CHL skills may be regarded as more subtle and complex [39]. This implies that health care providers may not recognize symptoms or may neglect signs of low CHL. With health care providers’ greater knowledge about HL and CHL’s seemingly greater importance than FHL in health encounters, the observed overestimation of individuals’ HL [40, 41] might decrease. Taking HL into account in health communication with refugees, for example by using clear communication [42] and teach-back [14], may further improve HEA and make HEA more useful to its participants.

Poor language skills are often used to explain communication problems between health care providers and migrants [10, 21, 36, 38, 43]. This is in agreement with our results that show that not using an interpreter increases the odds of communication problems during the HEA. Stigma and shame related to limited HL [18] may discourage people from asking questions when an interpreter is present. Further, the associations between low CHL and experiencing communication problems indicate the need for advocacy in the health authority to ensure provision of interpreters during the HEA and other health encounters.

### Limitations and strengths

This study has some important limitations. Subjective measures were used as the key outcomes, and such questions may be understood differently by different people, especially persons with different cultural and educational backgrounds [11, 13, 16]. Even if the HL scales were validity tested by cognitive interviews, more aspects of validity are needed to secure the quality of the HL scales across different populations. Further, the HL measures have not previously been used as self-assessing instruments.

Health literacy is intrinsically associated with an inadequacy in understanding information, which affects data collection. While language supporters were used to increase the quality and completeness of the data, there was still a large number of missing values in some items and thus in the indices. An attempt was made to address this problem in an appropriate way.

The use of retrospective questions about HEA and variables measuring HL at the time the participants fill in the form in the same analysis allows different interpretations. An alternative interpretation of the results is that the participants who perceived good quality of communication and/or usefulness of the HEA increased their HL in the HEA process. However, the present results are important because they give knowledge about refugees’ thoughts about HEA that may have an effect on which information they spread to others about HEA.

Older people, well-educated people and people with poor health may be underrepresented in the study, since these persons tend to participate in SFI to a lesser extent than others [44, 45]. However, a wide range of refugees were reached because most participate in SFI as a part of the program to which they are entitled by Swedish law, which should be seen as a strength. The use of translated questionnaires enabling collection of data from refugees with different countries of origin may also strengthen the quality of the study. The use of language supporters increased the quality of the communication and enabled answers from the relatively large proportion of illiterate people in the target group, which would otherwise have been excluded.

All SFI students fulfilling the inclusion criteria took part in the study. However, there were internal missing values for different questions. This may be considered a limitation for the multivariate analyses, which were based on all questions to be answered. However, the associations found in the multivariate analyses mainly appeared in the univariate analyses as well, where the number of participants was larger. For the index of comprehensive HL, internal missing values were handled in accordance with the index construction. The regression analyses showed the same results, independent of whether the individuals with missing answers were included in the analyses. Thus, the study results from which our conclusions are drawn seem to be independent of the internal missing values. In terms of having had the help of an interpreter, however, the results differed between the univariate and multivariate analyses, and the analyses should be interpreted with caution for this factor.

## Conclusion

A considerable proportion of refugees in Sweden had bad experiences of the communication in and usefulness of the HEA. Refugees’ experience indicates that a low level of CHL can act as a barrier to fulfilling the purposes of HEA. CHL may be of greater importance in that context than FHL. HL must be highlighted and acted upon in clinical praxis to increase the quality of HEA. Taking HL into account in health communication with refugees, for example by using clear communication [42] and teach-back [14], may improve HEA and make HEA more useful to its participants.

## Additional files

Additional file 1:
**Swedish functional health literacy scale ‐ English version.** (DOCX 58 kb) Additional file 2:
**Modified version of the HLS-EU-Q16.** (DOC 158 kb) Additional file 3:
**Questions about and dependent variables in the health examination with response categories.** (DOCX 13 kb) Additional file 4:
**Proportions of respondents with non-good experiences about the communication quality and the usefulness of the health examination** ^**a**^ **.** (DOCX 17 kb)
