# Supplementary material for: Health literacy and refugees’ experiences of the health examination for asylum seekers – a Swedish cross-sectional study
Source: BMC Public Health. 2015 Nov 23;15:1162. doi: 10.1186/s12889-015-2513-8 (PMC4657287; doi:10.1186/s12889-015-2513-8)
Supplement: Additional file 1: — Swedish functional health literacy scale ‐ English version. (DOCX 58 kb) [file 12889_2015_2513_MOESM1_ESM.docx]

**Additional file 1. Swedish functional health literacy scale ‐ English version**

| **Questions about how it is for you to take in information related to health, illness**  **and medical care.**  *Select the option on each line that best matches your answer.* | | | | | |
| --- | --- | --- | --- | --- | --- |
|  | Never | Seldom | Some­times | Often | Always |
| a. Do you think that it is difficult to read health infor­mation because the text is difficult to see (even if you have glasses or contact lenses)? |  |  |  |  |  |
| b. Do you think that it is difficult to understand word or numbers in health information? |  |  |  |  |  |
| c. Do you think that it is difficult to understand the message in health information? |  |  |  |  |  |
| d. Do you think that it takes a long time to read health information? |  |  |  |  |  |
| e. Do you ever ask someone else to read and explain health information? |  |  |  |  |  |
