# Supplementary material for: Health literacy and refugees’ experiences of the health examination for asylum seekers – a Swedish cross-sectional study
Source: BMC Public Health. 2015 Nov 23;15:1162. doi: 10.1186/s12889-015-2513-8 (PMC4657287; doi:10.1186/s12889-015-2513-8)
Supplement: Additional file 2: — Modified version of the HLS-EU-Q16. (DOC 158 kb) [file 12889_2015_2513_MOESM2_ESM.doc]

**Additional file 2. Modified version of the HLS-EU-Q16**

Developed by J.Wångdahl and L. Mårtensson based on the original version, HLS-EU Consortium (2012).

**Questions about health information**

| **Questions about how it is for you to find, understand and use information related to health, illness and medical care.** | | | | | |
| --- | --- | --- | --- | --- | --- |
| *Select the option on each line that best matches your answer.* | | | | | |
|  | Very easy | Easy | Difficult | Very difficult | Don’t know |
| a. How easy/difficult is it for you to find information on treatments of illnesses that concern you? |  |  |  |  |  |
| b. How easy/difficult is it for you to find out where to get professional help when you are ill (e.g. doctor, pharma­cist or psychologist)? |  |  |  |  |  |
| c. How easy/difficult is it for you to understand what your doctor says to you? |  |  |  |  |  |
| d. How easy/difficult is it for you to understand your doc­tor´s or pharmacist´s instruction on how to take a pre­scribed medicine? |  |  |  |  |  |
| e. How easy/difficult is it for you to judge when you need to get a second opinion from another doctor? |  |  |  |  |  |
| f. How easy/difficult is it for you to use information the doctor gives you to make decisions about your illness? |  |  |  |  |  |
| g. How easy/difficult is it for you to follow instructions from your doctor or pharmacist? |  |  |  |  |  |
| h. How easy/difficult is it for you to find information on how to manage mental health problems such as stress and depression? |  |  |  |  |  |
| i. How easy/difficult is it for you to understand warn­ings about behaviour (e.g. smoking, low physical activity and drinking too much)? |  |  |  |  |  |
| j. How easy/difficult is it for you to understand why you need health screenings (such as breast exam, blood sugar- or blood pressure test)? |  |  |  |  |  |
| k. How easy/difficult is it for you to judge if the informa­tion on health risks in the media is reliable (e.g. from TV or internet)? |  |  |  |  |  |
| l. How easy/difficult is it for you to decide how you can protect yourself from illness based on information in media (e.g. newspapers, leaflets and internet)? |  |  |  |  |  |
|  |  |  |  |  |  |
| *Select the option on each line that best matches your answer.* | | | | | |
|  | Very easy | Easy | Difficult | Very difficult | Don’t know |
| m. How easy/difficult is it for you to find out about activi­ties that are good for your mental well-being (e.g. medi­tation, exercise and walking)? |  |  |  |  |  |
| n. How easy/difficult is it for you to understand advice on health from your family members or friends? |  |  |  |  |  |
| o. How easy/difficult is it for you to understand informa­tion in the media on how to get healthier (e.g. from the internet, daily or weekly magazines)? |  |  |  |  |  |
| p. How easy/difficult is it for you to judge which everyday behaviour is related to your health (e.g. eating habits, exercise habits and drinking habits)? |  |  |  |  |  |
