# Supplementary material for: Health literacy and refugees’ experiences of the health examination for asylum seekers – a Swedish cross-sectional study
Source: BMC Public Health. 2015 Nov 23;15:1162. doi: 10.1186/s12889-015-2513-8 (PMC4657287; doi:10.1186/s12889-015-2513-8)
Supplement: Additional file 3: — Questions about and dependent variables in the health examination with response categories. (DOCX 13 kb) [file 12889_2015_2513_MOESM3_ESM.docx]

| **Additional file 3. Questions about and dependent variables in the health examination with response categories** | | |
| --- | --- | --- |
| **Dependent variable** | **Question** | **Response categories** |
|  | Have you participated in a free health check-up for asylum seekers? | Yes, before I got my residence permit Yes, after I got my residence permit Yes, but I don’t remember when No Don’t know |
|  | When did you do the health check-up for asylum seekers? | Less than 1 year ago Between 1 and 2 years ago More than 2 years ago |
|  | Was an interpreter present when you had the health check-up for asylum seekers? | Yes, No |
| **Quality of communication** | Did you understand what you were told when you did the health check-up for asylum seekers? | Yes, Partly, No, Don't remember |
|  | Could you talk about the health problem that you had when you did the health check-up for asylum seekers? |  |
|  | Could you ask the questions that you had when you did the health check-up for asylum seekers? |  |
|  | Did you get the answers to the questions you asked when you did the health check-up for asylum seekers? |  |
| **Receiving health care information** | Did you get information about… | Yes, No, Don't remember |
|  | … what rights asylum seekers have to health and medical care in Sweden, when you did the health checkup for asylum seekers? |  |
|  | …where you can go if you become sick in Sweden, when you did the health check-up for asylum seekers? |  |
|  | …where you can go for help in Sweden if you feel mentally unwell (for example, if you are very sad, stressed or worried), when you did the health check-up for asylum seekers? |  |
| **Receiving new knowledge** | Did you receive new knowledge that can contribute to improve your health, when you did the health check-up for asylum seekers? | Yes, Partly, No, Don't remember |
| **Receiving help** | Did you get help for the health problem that you had, when you do the health check-up for asylum seekers? |  |
