# Supplementary material for: Health literacy and refugees’ experiences of the health examination for asylum seekers – a Swedish cross-sectional study
Source: BMC Public Health. 2015 Nov 23;15:1162. doi: 10.1186/s12889-015-2513-8 (PMC4657287; doi:10.1186/s12889-015-2513-8)
Supplement: Additional file 4: — Proportions of respondents with non-good experiences about the communication quality and the usefulness of the health examination a . (DOCX 17 kb) [file 12889_2015_2513_MOESM4_ESM.docx]

| **Additional file 4. Proportions of respondents with non-good experiences about the communication quality and the usefulness of the health examination^a^** | | | | | |
| --- | --- | --- | --- | --- | --- |
|  | **Total study population** | **Functional health literacy** | | | |
|  | **Respondents/ response**  **category** | **Inadequate** | **Problematic** | **Sufficient** | **p-value** |
| **Agreement with different answer categories** |  | 238 | 78 | 80 |  |
|  | **number (%)** | **number (%)** | **number (%)** | **number (%)** |  |
| **Understood what was being told (n = 329)** |  |  |  |  | 0.304^b^ |
| No | 15 (5.1) | 12 (6.8) | 0 (0.0) | 3 (4.8) |  |
| Partly | 63 (21.4) | 13 (23.6) | 13 (23.6) | 15 (24.2) |  |
| Yes | 216 (73.5) | 130 (73.4) | 42 (76.4) | 44 (71.0) |  |
| **Could talk about health problems (n = 321)** |  |  |  |  | 0.154^b^ |
| No | 43 (14.8) | 27 (15.7) | 3 (5.4) | 13 (20.6) |  |
| Partly | 20 (6.9) | 13 (7.6) | 4 (7.1) | 3 (4.8) |  |
| Yes | 228 (78.4) | 132 (76.7) | 49 (87.5) | 47 (74.6) |  |
| **Could ask questions (n = 318)** |  |  |  |  | 0.055^c^ |
| No | 47 (16.3) | 32 (18.8) | 4 (7.1) | 11 (17.5) |  |
| Partly | 50 (17.3) | 29 (17.1) | 15 (26.8) | 6 (9.5) |  |
| Yes | 192 (66.94) | 109(64.1) | 37 (66.1) | 46 (73.0) |  |
| **Received answers to questions asked (n = 299)** |  |  |  |  | 0.070^c^ |
| No | 44 (16.4) | 26 (16.3) | 7 (14.0) | 11 (18.6) |  |
| Partly | 51 (19.0) | 33 (20.6) | 14 (28.0) | 4 (6.8) |  |
| Yes | 174 (64.7) | 101 (63.1) | 29(58.0) | 44 (74.6) |  |
| **Quality of communication (n = 275)** |  |  |  |  | 0.429^c^ |
| Low quality | 161 (64.4) | 56 (38.9) | 16 (32.7) | 17 (29.8) |  |
| High quality | 89 (35.6) | 88 (61.1) | 33 (67.3) | 40 (70.2) |  |
| **Having received information about** |  |  |  |  |  |
| **… asylum seekers’ rights to health (n = 306)** |  |  |  |  | 0.488^c^ |
| No | 120 (44.0) | 79 (46.7) | 19 (40.4) | 22 (38.6) |  |
| Yes | 153 (56.0) | 90 (53.3) | 28 (59.8) | 35 (61.4) |  |
| **…where to go if one becomes sick in Sweden**  **(n = 324)** |  |  |  |  | 0.730^c^ |
| No | 67 (22.9) | 43 (24.4) | 12 (21.8) | 12 (19.7) |  |
| Yes | 225 (77.1) | 133 (75.6) | 43 (78.2) | 49 (80.3) |  |
| **…where to go in Sweden if one is mentally unwell (n = 306)** |  |  |  |  | 0.799^c^ |
| No | 153 (54.6) | 92 (55.4) | 31 (56.4) | 30 (50.8) |  |
| Yes | 127 (45.6) | 74 (44.6) | 24 (43.6) | 29 (49.2) |  |
| **Receiving little health care information (n = 206)** |  |  |  |  | 0.644^c^ |
| Little | 102 (41.8) | 65 (43.9) | 18 (40.9) | 19 (36.5) |  |
| Much | 142 (58.2) | 83 (56.1) | 26 (59.1) | 33 (63.5) |  |
| **Receiving new knowledge (n = 301)** |  |  |  |  | 0.520^c^ |
| No | 16 (41.9) | 74 (44.3) | 22 (40.7) | 20 (35.7) |  |
| Partly | 59 (21.3) | 32 (19.2) | 15 (27.8) | 12 (21.4) |  |
| Yes | 102 (36.8) | 61 (36.5) | 17 (31.5) | 24 (42.9) |  |
| **Receiving help (n = 316)** |  |  |  |  | 0.113^c^ |
| No | 73 (25.4) | 48 (28.1) | 16 (28.6) | 9 (15.0) |  |
| Partly | 88 (30.7) | 50 (29.2) | 18 (32.1) | 20 (33.3) |  |
| Yes | 126 (43.9) | 73 (42.7) | 22 (39.3) | 31 (51.7) |  |
| ^a^Missing data not included; ^b^Fischer´s exact test; ^c^Chi-square test n ( %) = number and proportions of participants; significant differences p < 0. 05 are printed in bold. | | | | | |
